# Data Maturity Assessment in Long-Term Care: Mixed Methods Study

**DOI:** 10.2196/93162

**Published:** 2026-08-19

**Authors:** Suleyman Bouchmal, Katya Sion, Pepijn Janssens, Manouk ten Thij, Jan Hamers, Sil Aarts

**Affiliations:** 1 Living Lab in Ageing and Long-Term Care, Maastricht University, Duboisdomein 30, Maastricht, 6229 GT, The Netherlands, 31 43 388 2222; 2 Department of Health Services Research, CAPHRI Care and Public Health Research Institute, Faculty of Health Medicine and Life Sciences, Maastricht University, Maastricht, The Netherlands; 3 Zorggroep Meander, Landgraaf, Limburg, The Netherlands

**Keywords:** data maturity, long-term care, data-informed care, interprofessional collaboration, organizational culture

## Abstract

**Background:**

The use of data to support decision-making and primary processes is central to establishing data-informed care. Yet, data remain underutilized for quality improvement in long-term care (LTC). Data maturity reflects an organization’s capability to use data, for example, to guide strategic objectives.

**Objective:**

This study aimed to assess the data maturity of LTC organizations and evaluate the opinions and ideas of different stakeholders (eg, client representatives, care professionals, and data and IT specialists) within these organizations regarding their data maturity.

**Methods:**

An exploratory mixed methods study was conducted in 3 Dutch LTC organizations. Quantitative data were collected using a 7-domain data maturity assessment comprising 77 items with 3 response options (applicable, not applicable, or in progress), which was completed through consensus-based scoring. Each organization established an interprofessional community of practice (CoP), consisting of 5 to 6 purposively recruited stakeholders representing care professionals, managers, client representatives, IT and data specialists, and researchers. Qualitative data were collected through consensus meetings with the same CoP stakeholders to elaborate on and contextualize their data maturity assessments. Quantitative data were analyzed descriptively, while qualitative data were analyzed using hybrid thematic analysis.

**Results:**

Findings highlighted that LTC organizations currently operate at a low data maturity level. Domains regarding strategy, governance, and data quality were low yet consistent across organizations, whereas variability and substantial gaps were mainly present in the domain regarding leadership and culture. The consensus meetings with 15 stakeholders in the CoPs (mean age 43, SD 11 y; mean organizational work experience 10, SD 9 y) identified 4 overarching themes: (1) the absence of a clear vision for data-informed care reflects low data maturity, (2) a data culture is a prerequisite for continuous learning and improvement, (3) innovation and transformation are constrained by limited interdisciplinary engagement and ineffective communication, and (4) sustainable data use is hindered by infrastructural capacity and cultural readiness.

**Conclusions:**

This study contributes to the limited evidence on data maturity in LTC, revealing generally low levels of data maturity across organizations. Advancing data-informed care requires integrated strategies that align technical, cultural, and interprofessional conditions. Future research should adopt longitudinal designs and broader stakeholder involvement to strengthen data-informed care in LTC.

## Introduction

Long-term care (LTC) for older adults involves integrated health and social care for individuals with complex, often chronic needs across community and residential settings [1]. Ensuring quality of care in these settings requires combining evidence-based practice and clinical expertise with an understanding of clients’ preferences and values [2,3]. Across client, staff, and organizational levels, multimodal data are routinely collected to inform the understanding of quality of care. Despite their potential, data from questionnaires, observations, conversations, electronic health records (EHRs), and technology (eg, wearables) remain underutilized [4]. Bridging the gap between data collection and practical application to generate actionable insights is essential for embedding continuous quality improvement in care delivery.

Incorporating data into daily care practices enables data-informed care as a supportive element in routine practice. Data-informed care combines quantitative data with qualitative insights, including the experiences and knowledge of clients, their relatives, and care professionals [5,6]. Consequently, data facilitate reflection on and learning from past events, supporting data-informed decision-making [7]. Yet, to serve this purpose, data must be rendered usable and interpretable. Recent developments in LTC include the implementation of business intelligence (BI) solutions to centralize data and improve interoperability across data systems [8]. BI involves the process of analyzing, transforming, and integrating data into actionable insights to support various tasks (eg, employee deployment and strategic planning) [9]. However, the literature indicates that organizational barriers such as the absence of a clear vision and strategy for data use, data fragmentation caused by decentralized systems, and limited data literacy among care professionals can impede the effective use of data within LTC organizations [6].

The introduction of data-informed care depends on an organization’s broader capacity for improvement. A growing body of research emphasizes the importance of organizational capacity to overcome barriers that hinder development and quality improvement [10,11]. The concept of organizational maturity refers to the organization’s ability (ie, readiness, willingness, and completeness) to work toward its goals [12,13]. Derived from this, data maturity refers to the organization’s capability to systematically use data, for example, by leveraging data insights to support decision-making and achieve strategic objectives [14]. The assessment of data maturity requires the evaluation of several domains, such as IT infrastructure, organizational processes, and employees’ competencies in data use [15]. Overall, the level of data maturity provides an understanding of the status quo and how organizations might evolve in their ability to manage, integrate, and apply data to achieve continuous improvement [16].

To date, scientific literature regarding data maturity assessments (DMAs) in care organizations is scarce or even absent. Hence, the relationship between an organization’s data maturity and its ability to monitor, evaluate, and enhance the quality of care remains largely unexplored. To gain insight into how LTC organizations use data and the domains (eg, IT architecture, leadership, data quality) in which improvements are needed, a systematic evaluation of their capabilities is needed. The aim of this study was to assess the data maturity of LTC organizations in the Netherlands and evaluate how different stakeholders (eg, client representatives, care professionals, and data and IT specialists) within an organization view the organization’s data maturity.

## Methods

### Study Design

This exploratory cross-sectional study used a convergent mixed methods design [17]. Quantitative data were collected using the DMA, while qualitative data were simultaneously collected through 3 consensus meetings. Consensus meetings are structured group discussions in which interprofessional stakeholders discuss a predefined topic and collectively work toward reaching agreement on a shared decision. Both data sources were collected concurrently from May to June 2024 within the same sessions, analyzed separately, and integrated during the interpretation phase, with the qualitative findings used to contextualize and explain the quantitative DMA results.

### Setting and Participants

The study was conducted in 3 Dutch LTC organizations participating in the Limburg Living Lab in Ageing and Long-Term Care (*Academische Werkplaats Ouderenzorg Limburg* [AWO-L]), a formal collaboration between 9 LTC organizations and 4 educational institutions spanning vocational, bachelor’s, and master’s education. The 3 participating LTC organizations were purposively selected from the Living Lab based on their ability and willingness to participate in an overarching 4-year participatory research study on data-informed care and their commitment to support the implementation and evaluation of organization-specific data use cases [18]. As this study was embedded within the 4-year research study, participating organizations were required to commit the necessary organizational resources (eg, staff time and meeting facilities) and engage in sustained collaboration throughout the project. All 3 organizations had existing communities of practice (CoPs) aimed at fostering learning and quality improvement related to data-informed LTC. A CoP is an interprofessional collaboration of individuals aiming to generate, apply, and disseminate knowledge among stakeholders [19]. Each participating LTC organization nominated stakeholders for the CoP based on their role, expertise, and involvement in care processes, organizational decision-making, or data-related activities, ensuring representation from diverse perspectives. The nominated stakeholders were free to decide whether they wanted to participate in the CoPs. The aim was to establish a committed, interprofessional group of stakeholders willing to collaborate over a 4-year period on the development, implementation, and evaluation of data-informed solutions for organization-specific use cases. Each CoP has been in place for approximately 6 months and consists of a heterogeneous group of 5 to 6 stakeholders, including care managers, nurses, client representatives, IT and data specialists, and researchers.

### Data Collection

#### DMA

In the Netherlands, the national network “RegioPlus,” together with ActiZ and Vilans, has developed a prevalidated DMA for the health care sector [15]. The DMA has undergone preliminary testing to assess its clarity, feasibility, and initial reliability but has not yet been subjected to a comprehensive validation procedure. However, this assessment is completed annually by several hundred organizations at the national level. All stakeholders in the 3 CoPs were provided with a paper version of the DMA.

The DMA comprises three parts: (1) part 1 focuses on organizational characteristics; (2) part 2 addresses 7 domains related to an organization’s data maturity; and (3) part 3 examines current organizational challenges and needs. In line with the aim of the study, the focus was placed on part 2 of the DMA, in which 77 items are categorized into 7 domains—as presented in Table 1. The response options for each item are as follows: (1) “applicable,” (2) “not applicable,” and (3) “not applicable, but in progress.”

**Table 1.** Overview of 7 domains with short descriptions and item examples.

Domains (number of items)	Short description	Item examples
Leadership and culture (14)	Items related to values, organizational leadership, and cultural aspects supporting data use	Leaders are curious about the meaning of data for the organization. Experiments are encouraged. Working with data is assigned to a separate Business Intelligence (BI) entity.
Strategy and governance (10)	Items concerning strategic alignment, decision-making structures, and governance of data.	Policy goals are quantified by theme and domain to increase manageability. Data supports decision-making. In collaboration with partners, continuous work is done on data-informed policy research using data science technology.
Process-oriented work (10)	Items on embedding data use into organizational processes and workflows.	The Data Protection Officer is involved at the start of experiments. In policy and decision-making processes, the addition of data insights is a standard component.
Definitions and data quality (11)	Items addressing consistency of definitions, standards, and data quality management.	Coordination on data management and shared definitions occurs between departments. Data flows are monitored by tools using organization-wide data technologies (such as FAIR [Findable, Accessible, Interoperable, and Reusable] principles, linked data).
Architecture and tooling (10)	Items regarding IT infrastructure, (data sciences) tools, and support for data use.	Technical and functional integrated IT architecture is considered a critical “asset” for the organization, with appropriate resources and governance. A central environment (data warehouse) continuously collects internal, external, and public data selected for relevance.
Employee skills and knowledge (12)	Items on staff competencies, training, and knowledge for working with data.	Care professionals participate in multidisciplinary teams where, with the help of data insights (especially through integrated dashboards), concrete issues are interpreted and actionable steps are provided. Care professionals are able to provide professional input for the development of predictive models.
External collaborations (10)	Items regarding partnerships and collaborations with external stakeholders for data use.	The organization collaborates with the patient or client, for example, via secure email or a client portal. Decisions are made jointly with other organizations based on predictive insights from combined data.

#### Consensus Meetings

While administering the DMA within the CoPs, consecutively during the same session with the same CoP stakeholders, consensus meetings were conducted to achieve collective agreement through group dialog for each response to the DMA. Thus, per organization, 1 DMA was completed. Given the diverse backgrounds of the CoPs’ members who had been involved over the past half-year, the discussions were organized with an emphasis on open communication. The moderator encouraged participation from all stakeholders and facilitated the discussion until the group reached a shared rating for each statement [20].

Each consensus meeting was guided by a semistructured approach, with questions designed around the topics covered in 7 domains of the DMA. The consensus meetings were facilitated by 4 researchers. The first author (SB) served as the primary moderator, while 2 researchers (PJ and MtT) asked follow-up and probing questions to further explore stakeholders’ responses. The fourth researcher (KS or SA) observed the discussions and documented field notes throughout the sessions. The questions primarily explored stakeholders’ experiences and understanding of the 77 individual DMA items (ie, data from the primary care processes is trustworthy, or strategic objectives are determined by data insights on the organizational level). Each DMA item was initially completed individually by the stakeholders within the CoPs. Immediately thereafter, the item was discussed as a group to substantiate the individual assessments and reach consensus regarding its applicability. This iterative process was repeated for all 77 items. Completing the DMA and the accompanying consensus meeting required approximately 3 hours per CoP. As the response options of the DMA consisted of 3 choices, the dialogue focused on the argumentation of the group. Specifically, if the CoP argued that a certain statement was (not) applicable to their organization, the research group was interested in examples of how or why this was (not) embedded within the organization. As these discussions took place within established CoPs, stakeholders had already collaborated over an extended period of time, fostering mutual trust and open dialogue across professional roles. Throughout the discussions, the facilitator actively encouraged balanced participation to ensure that all perspectives were heard. By sharing their experiences and knowledge, stakeholders contributed to a collaborative understanding that enriched the discussion and supported group cohesion [21]. The consensus meetings were audio-recorded and transcribed verbatim using a speech-to-text model developed internally within the AWO-L [22].

### Data Analysis

#### Stakeholder Characteristics

During the informed consent process, additional information was collected from each stakeholder, including age, gender, job title, and years of experience in their current profession. Descriptive analyses were conducted to summarize the stakeholders’ demographics, such as mean age, SDs, and range.

#### DMA

Data analysis was focused on the 7 domains of the DMA. These 7 domains covered 77 items (range 10‐14 items per domain). Responses from the care organizations were entered into SPSS (IBM Corp). Frequency distributions were analyzed to assess how the responses were distributed across the 3 response options (“applicable”, “not applicable,” and “not applicable, but in progress”).

#### Consensus Meetings

The transcripts were reviewed for accuracy and uploaded to MAXQDA (Verbi Software) [23]. The first author thoroughly familiarized himself with the data by repeatedly reading all transcripts, enabling a deeper understanding of the content [24]. As this study respected the 7 domains for the DMA as a framework, a hybrid thematic analysis was conducted. Initial coding was guided deductively by the predefined DMA domains and the consensus meetings topic guide, while inductive coding was subsequently used to identify emerging themes and patterns that extended beyond the predefined framework within these domains [25]. Codes were iteratively refined and grouped into overarching themes through discussion within the research team. A step-by-step approach guided the qualitative analysis. First, within each of the 7 DMA domains, stakeholders’ responses were systematically reviewed. Meaningful segments of text were identified and annotated with labels that captured the essence of stakeholders’ experiences and perspectives on data maturity [26]. Second, the labels with text segments were examined for patterns and grouped into broader categories regarding their coherence within the 7 domains of the DMA, often referred to as axial coding [27]. This step involved summarizing recurring ideas from the stakeholders and identifying conceptual similarities within the DMA domains. Third, these grouped codes were synthesized into overarching themes that captured the most salient insights across the consensus meetings. The themes were refined through comparison with the original data, ensuring that they accurately represented stakeholders’ views while offering deeper interpretative value beyond the quantitative results. While 3 authors (SB, PJ, and MtT) conducted the initial coding collaboratively, all codes and emerging themes were reviewed and discussed with the whole research team. During the entire coding process, all authors reviewed the resulting themes through iterative discussions to reach consensus on the interpretation and content of the themes [28].

### Ethical Considerations

The study protocol was approved by the Faculty of Health, Medicine and Life Sciences Research Ethics Committee of Maastricht University (FHML-REC/2024/055). Stakeholders were informed about the study’s purpose in advance via email. The email outlined the study’s objectives and provided essential details regarding the consensus meeting and the DMA, including the location, date, and time. Participation was strictly voluntary, and informed consent was obtained. Stakeholders were free to withdraw from the study at any time, for any reason, without providing a reason. To ensure the anonymity of stakeholders, no names or organizational details were recorded.

## Results

### Stakeholder Demographics

A total of 15 stakeholders participated in the study, 5 from each of the 3 CoPs (organizations A, B, and C). Stakeholders had a mean age of 43 (SD 11; range 23‐63) years and a mean work experience of 10 (SD 9) years within their organizations, representing a range of professional disciplines and organizational perspectives. The descriptive characteristics of the stakeholders are presented in Table 2.

**Table 2.** Descriptive characteristics of stakeholders from 3 long-term care (LTC) organizations (N=15).

Characteristics	Values
Mean age in years (SD; range)	43 (11; 23‐63)
Mean years of work experience (SD; range)	10 (9; 0^a^-40)
Sex, n (%; females)	9 (60)
Profession (n)	
Health care professionals^b^	6
Business intelligence (BI) specialists^c^	3
Members client council	2
Managers	3
Researcher	1

a Stakeholder employed for <3 months before the assessment.

b Health care professionals included 4 nurses, 1 specialist in involuntary treatment, and 1 specialist in client well-being.

c BI specialists included 2 BI architects and 1 specialist in data storage and exchange.

### DMA

**Figure 1.**
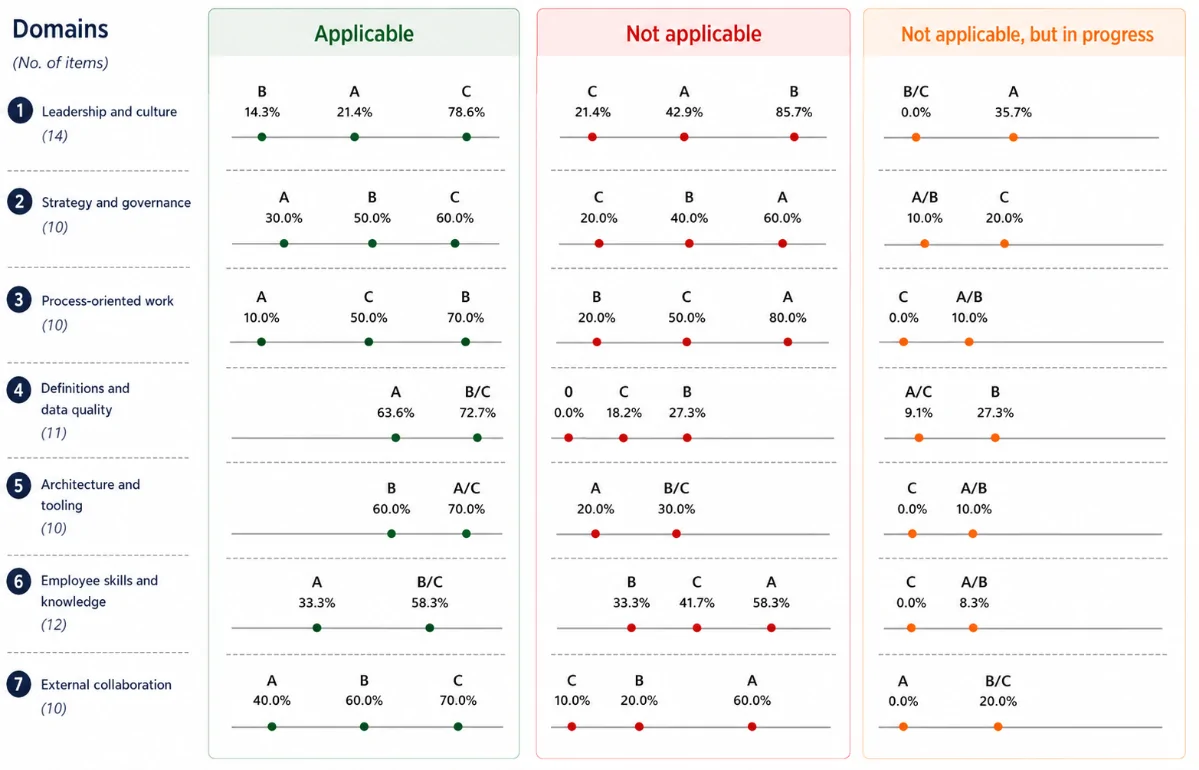
Ranges per domain across organizations by response option. The figure presents the distribution (in percentages) of responses across the 7 domains and 3 response categories: applicable (the item is applicable within the organization), shown in green; not applicable (the item is not applicable within the organization), shown in red; and not applicable, but in progress (the item is not applicable yet, but implementation is underway), shown in orange. Within each domain and response category, organizations A, B, and C are arranged from left to right in ascending order of their percentage scores. The position of each dot on the horizontal line represents the percentage score (0%-100%). The horizontal line represents a scale ranging from 0% (left) to 100% (right), and each dot represents an organization’s percentage score on the scale. A, B, and C denote organizations A, B, and C, respectively.

Figure 1 presents the number of responses for each option, categorized by domain and organization. The number of responses is normalized. For the first response option (applicable), while considering the unequal distribution of items across domains, the mean count was 6 across all organizations. This indicates a comparable yet relatively low-to-average level of applicability reported across domains, despite the unequal number of items per domain. However, variability differed between organizations. Specific domains, such as strategy and governance, definitions and data quality, and architecture and tooling, showed a narrow range, suggesting greater consistency across organizations regarding these themes. Other domains, such as leadership and culture and process-oriented work, showed a wider range, indicating greater differences across organizations.

Regarding the second response option (not applicable), the mean count for all items per domain was 4 across all organizations. This indicates that several items are not yet established, reflecting limited integration of the assessed 7 domains. Domains that scored similarly across organizations were definitions and data quality, architecture and tooling, and employee skills and knowledge. More differences were found in domains such as leadership and culture and process-oriented work, indicating that responses within these domains were more dispersed.

Regarding the last response option (not applicable, but in progress), the mean count for all items per domain was 1, indicating that the organizations had relatively little work in progress concerning the items across the assessed domains. All domains showed a very narrow range for all organizations, except for leadership and culture.

### Consensus Meetings

#### Overview of Themes

Thematic analysis of the stakeholder’s responses revealed 4 prominent themes, all providing more in-depth arguments and evidence to support the answers given in the DMA. The four themes were as follows: (1) the absence of a clear vision for data-informed care reflects low data maturity, (2) a data culture is a prerequisite for continuous learning and improvement, (3) innovation and transformation are constrained by limited interdisciplinary engagement and ineffective communication, and (4) sustainable data use is constrained by infrastructural capacity and cultural readiness. The 4 overarching themes, including their underlying axial codes, are presented in Table 3.

**Table 3.** Overview of themes and axial codes.

Themes	Axial codes
T1—the absence of a clear vision for data-informed care reflects low data maturity	Lack of engagement in data strategies Unclear accountability for data initiatives Ambiguity in data-related goals and priorities at the organizational level Failure to link data vision to quality of care Disconnection between vision and reality, practice, and primary processes Top-down approaches without input from primary processes Limited awareness of data potential among leaders Lack of organizational direction for data use Underdeveloped data governance structures
T2—a data culture as a prerequisite for continuous learning and improvement	Experimentation with small-scale data projects Need for structural follow-up on pilot projects Growing awareness of data relevance in care processes Slow but visible improvements in staff’s data literacy Growing awareness of data relevance in quality of care
T3—innovation and transformation constrained by limited interdisciplinary engagement and ineffective communication	Underutilized (potential of) interdisciplinary learning Minimal to no involvement of data specialists in care teams IT is viewed as separate from care delivery and primary processes Siloed working practices hinder collaboration Lack of shared language between disciplines
T4—sustainable data use is hindered by infrastructural capacity and cultural readiness	Little investments in data usage (infrastructure, budget, etc) Training available is not tailored or adequate Fragmentation in data tools and projects Organization culture is insufficient for data usage Low trust and awareness in digital solutions among professionals Reactive rather than proactive data practices Data usage driven by necessity rather than strategy Poor communication across organizational levels Inconsistent communication from management

#### The Absence of a Clear Vision for Data-Informed Care Reflects Low Data Maturity

Many of the stakeholders expressed their concerns during the consensus meetings about the absence of a clear vision and strategy for data-informed care within their organization. They noted that managers, directors, or chief executive officers had not provided any tangible objectives, leaving staff with a feeling of lacking clear direction.

*I have not seen any references towards data-informed care within the strategy of the organization.*

[Org. B]

*We don’t have a strategy to implement data-informed care. This is only briefly mentioned in a plan for this year within another plan [technology in care].*

[Org. C]

Moreover, stakeholders mentioned that there is uncertainty among staff about what the future holds. Efforts to advance data-informed care should be initiated by top-level management, but stakeholders noted that this has yet to be recognized in practice.

*… the management team are the leaders, but I think they are not the same as the initiators. The initiative to work with data should first come from the board of directors. However, this is not the case, yet.*

[Org. A]

*As a result of leaders lacking relevant insights, we are often confronted with predetermined goals that do not align with our department’s needs.*

[Org. B]

The absence of a structured budget further reinforced perceptions of a lack of vision and strategy; according to the stakeholders, there is currently no financial support to drive these efforts.

*We want to experiment with data to improve data-informed care, but the current budget for experimentation is reaching its limits.*

[Org. A]

*A structural budget would really help—especially to bring in the right people who can actually move data-informed care forward.*

[Org. C]

#### A Data Culture as a Prerequisite for Continuous Learning and Improvement

Stakeholders noted that organizations are making gradual progress in strengthening the data culture, indicating that awareness regarding data is growing. Several aspects were highlighted, including improved staff involvement, well-adapted data systems (eg, EHRs) that support health care professionals in their daily tasks, and the introduction of data dashboards (ie, steering information). The inclusion of diverse stakeholders has fostered greater awareness and engagement with data, highlighting its importance in everyday work practices.

*We are aware and interested in the value of data-utilization, because a lot of data is used in yearly reports, such as data about clients, staff, and finances.*

[Org. B]

*Working with data is becoming increasingly important. New care managers have grown up with data or have used it in their previous roles. These managers are clear about how they want to utilize data in their teams.*

[Org. C]

Stakeholders observed that the data systems used by staff are becoming more reliable, partly due to the implementation of standardized instruments. For example, clinometric questionnaires in EHRs were valued for reducing variability and errors in documentation, while user-friendly functions were seen as supportive of daily work.

*In the open text fields, there’s still a wide variety in how data is recorded. However, when using clinometry or other standardized instruments, the input is more consistent and self-explanatory. It’s difficult to make mistakes with those, and everyone knows when and how to use them.*

[Org. A]

*… EHRs provide comprehensive client information, including care plans and progress tracking, which support our day-to-day work.*

[Org. B]

Dashboards were also recognized as a promising tool for providing overarching insights and making data more accessible. It was mentioned that data dashboard supports to introduce steering information, which can be acted upon. However, stakeholders mentioned that access remained restricted to a small group of users, limiting their organizational impact.

*With limited access, only a very small group is truly capable of working with BI independently. However, these could support the whole organization.*

[Org. B]

*By providing insights into departmental performance, dashboards help to make data more understandable.*

[Org. C]

However, some stakeholders also mentioned that current data usage is primarily ad hoc and concentrated in departments such as human resources and finance (eg, related to reports based on financial or quantitative outcomes). However, stakeholders expected that data would be most valuable when integrated into everyday care delivery.

*It is more ad hoc and reactive. In that sense, there is no real step-by-step plan to work with data.*

[Org. B]

*Only a small part of the organization actively utilizes data—primarily location and care managers, as well as staff in departments like finance and HR.*

[Org. C]

#### Innovation and Transformation Constrained by Limited Interdisciplinary Engagement and Ineffective Communication

According to the stakeholders, the potential for innovation through data-informed care is constrained by fragmented initiatives and a lack of effective, strategic communication across departments. Stakeholders noted that projects were often duplicated within or between LTC organizations and poorly communicated.

*In one department, they’ve just invented the wheel—thanks to an excellent graduation project from an intern, things are going well there. But in the very same organization, even just a floor above or below, another department has no idea it’s happening and ends up starting the exact same project from scratch… Ask the management, how many pilots are running? They have no idea.*

[Org. A]

*I believe the organization could substantially enhance its communication regarding data-informed care, as communication about this is largely absent.*

[Org. B]

Stakeholders noted that a more integrated approach, in which information, lessons learned, and support structures are systematically shared, could greatly enhance organizational learning and innovation capacity. In particular, the involvement of IT and BI specialists was viewed as critical, as they possess expertise in data management and system infrastructure, which is necessary to support data-informed initiatives.

*With support from IT, we’d have the right data at hand to see what’s already been done and how we can build on it. If projects overlap, sharing insights would help us learn from each other and avoid repeating work.*

[Org. A]

*They [IT/BI specialists] are required to participate in interprofessional projects and starting up different initiatives. All new areas or initiatives should start to ask regularly for support with BI-tools.*

[Org. C]

#### Sustainable Data Use Is Hindered by Infrastructural Capacity and Cultural Readiness

Stakeholders described persistent structural and cultural barriers that hinder sustainable data use in LTC organizations. Despite increasing recognition of the value of data-informed care, many stakeholders observed that data practices are not yet systematically embedded in daily workflows. A recurring issue was the lack of staff training, especially among care professionals, and low capacity building (ie, the use of resources and investments in data infrastructure) for introducing data-informed care. Although dashboards and data tools were often available, insufficient attention was paid to their implementation, usability, and integration into care processes.

*It’s not just about the tools. How do you train users, how do you collect feedback, and how do you ensure dashboards are implemented in a way that makes sense to care professionals? Those are the questions we should be asking ourselves.*

[Org. B]

*… it becomes clear that the execution or implementation of data initiatives is weakening, because they [managers] do not consider how it fits in work processes.*

[Org. C]

There was a shared perception that data initiatives often lacked a bottom-up approach, as participants noted that they were often implemented in a top-down manner. While stakeholders considered it desirable to involve a diverse group in this process, such as care professionals and even client representatives, they expressed concerns that care professionals were not adequately involved in implementation processes, nor were they meaningfully informed about the value and purpose of data use.

*The implementation process is often driven by a selected group, which is often not representative of all care professionals.*

[Org. A]

*I doubt that health care professionals are really been included in the implementation process. There hasn’t been much communication about why or how data should be used.*

[Org. B]

Although some stakeholders observed a growing interest in data use, they emphasized that without clear structures, resources, and bottom-up involvement, this awareness would remain superficial. The absence of visible benefits reinforced perceptions of data use as compliance-driven rather than a meaningful contributor to care improvement. Since the added value of data-informed care has not yet been emphasized within the organizations, stakeholders continued to perceive data use primarily as an administrative burden when reflecting on barriers to foster a data culture.

*I’m not sure all data is actually being applied well, but there’s definitely more awareness and interest in what data could contribute to our processes.*

[Org. A]

*They [health care professionals] may continue to perceive it as an administrative burden, as the added value is neither recognized nor communicated within the organization, resulting in limited interest.*

[Org. C]

### Synthesis

The discussions conducted during the consensus meetings enabled stakeholders to collectively reflect on and substantiate their DMA assessments, thereby providing valuable context for interpreting the quantitative findings. As a result, the quantitative and qualitative findings were highly complementary. Lower maturity in the domains of leadership and culture, and strategy and governance was reflected in stakeholders’ descriptions of an absent organizational vision, unclear responsibilities, limited leadership engagement, and underdeveloped governance structures for data-informed care. Similarly, lower scores in architecture and tooling were supported by stakeholders’ experiences of fragmented data tools, limited interdisciplinary collaboration with IT and BI specialists, and insufficient infrastructural capacity to support routine data use. Conversely, the relatively higher maturity observed in definitions and data quality aligned with stakeholders’ experiences of standardized documentation practices, the implementation of structured assessment instruments, and growing confidence in the quality of routinely collected data. Across all organizations, stakeholders further emphasized that, despite increasing awareness of the value of data-informed care, current data use remained largely reactive and concentrated within departments such as finance and human resources, rather than being systematically embedded in routine care. Together, the quantitative and qualitative findings indicate that, while important foundations for data-informed care are emerging, organizational leadership, governance, interdisciplinary collaboration, and infrastructural capacity remain key areas requiring further development to support its sustainable implementation.

## Discussion

### Principal Findings

The aim of this study was to assess the data maturity of several LTC organizations in the Netherlands and to evaluate how different stakeholders within an organization view the organization’s data maturity. Overall, the findings show that the data maturity in LTC organizations is low. Several domains of the DMA, such as strategy and governance, definitions and data quality, and architecture and tooling, consistently reflect low maturity across organizations, indicating structural, organization-wide limitations herein. In contrast, leadership and culture show substantial variability across organizations. In addition, stakeholders from LTC organizations emphasized that the absence of a clear organizational vision for data-informed care (eg, a formal document outlining the organization’s vision and strategy for data use) and limited communication about it within the organization reflect a low level of data maturity.

Various important factors that support data maturity in LTC organizations, including the need for clear leadership and an existing data-culture, are closely linked to the presence or absence of an organizational vision for data-informed care. When this vision is actively shaped (eg, stakeholders’ involvement and effective communication) and supported within the organization, data practices are more likely to become part of daily work [29]. As underscored in the literature, this study highlights a lack of clear leadership and shows that care staff often experience uncertainty regarding the purpose and value of data in their daily work [30]. Organizations where staff promote data use and support interprofessional collaboration demonstrate early signs of organizational learning and innovation [31]. The current study reveals that the involvement of IT and BI specialists remains largely disconnected from primary care processes. Although technical infrastructure is in place, the expertise required to translate raw data into meaningful insights regarding care provision is frequently siloed or absent, which limits shared understanding and reduces the ability of care teams to effectively work with the data [32]. For example, within the participating organizations, lower maturity in the architecture and tooling domain reflects an organizational environment in which routinely collected data cannot be readily integrated, accessed, and translated into actionable information to support care processes and decision-making. In practice, this may be characterized by fragmented information systems, limited interoperability between applications, restricted data accessibility, and challenges in integrating data across organizational systems. Although data may be routinely collected, the underlying technical infrastructure may not sufficiently support its efficient reuse for quality improvement, organizational learning, and data-informed care. Consequently, many data-related initiatives (eg, projects to use data for quality improvement) remain confined to isolated pilots, with limited alignment to broader organizational learning structures that support continuous improvement, thereby reinforcing a fragmented and inconsistent data landscape [33].

Data maturity is intertwined with concepts such as data management and data governance; all are essential to establish effective data stewardship, quality assurance, and control-mechanisms within organizations [34]. Data governance, in particular, is often closely aligned with an organization’s broader strategy and vision, influencing factors such as data quality, the perceived usefulness of information systems, and the effectiveness of data dissemination [35]. Because stakeholders consider an organizational vision a foundational prerequisite, data governance structures may also be insufficiently developed within these organizations. When reflecting on existing data management frameworks, such as the Data Management Body of Knowledge (DAMA-DMBOK), these emphasize the importance of structured governance, standardized processes, and clearly defined roles [36]. While such frameworks offer valuable conceptual guidance (eg, requirements for governance and architecture) to support data-informed care, the findings of this study suggest that these frameworks alone may not fully capture the uniqueness of the LTC setting, as stakeholders underscore the low cultural readiness and limited infrastructural capacity. LTC organizations typically operate with limited technical expertise, fragmented governance structures, varying levels of digital literacy among staff, and persistent resource constraints, indicating that a different approach might be required to implement data-informed care [6]. For instance, the DAMA-DMBOK framework assumes the availability of dedicated roles, organizational stability, and functional integration across departments; all conditions that might be lacking in LTC organizations. Furthermore, due to inconsistent data standards, the informal distribution of key responsibilities, and underdeveloped strategic alignment around data use, the implementation of comprehensive data use in practice remains challenging [37].

As the results of this study indicate, supporting data-informed care in LTC requires more than the implementation of digital tools and data infrastructures. Therefore, the paradigm of the learning health system provides an understanding, as it conceptualizes the process of transforming data from routine care into actionable knowledge by integrating data, knowledge, and practice in a continuous learning cycle [38]. As stakeholders highlight the importance of fostering a data culture to support continuous learning and quality improvement, the learning health system provides a complementary perspective by emphasizing continuous feedback, reflection, and adaptation as integral to everyday practice [39]. In a so-called socio-technical view, data maturity emerges through the dynamic interaction of infrastructure, people, and organizational culture [40]. This implies that policy from within the organization (formulated, eg, by management, chief executive officers, and policymakers) should also address organizational and professional conditions rather than digitalization alone, reflecting stakeholders’ reported constraints related to infrastructural capacity and cultural readiness. However, while Dutch and European policy initiatives, including the European Health Data Space, are emerging in response to the need to improve data use and data-informed care [41,42], translating these ambitions to the LTC context is not straightforward. LTC operates within a distinct ecosystem (eg, characterized by chronic care, diversity in resources, and reliance on informal caregivers) [43], requiring policies at the national level (eg, by the government) that acknowledge contextual barriers such as staffing capacity, governance fragmentation, and providing care for some of the most fragile people in our societies [44].

To our knowledge, this study is the first to provide insights into data maturity in LTC. It offers a comprehensive overview of organizational variation across key domains, supported by input from a diverse range of stakeholders. However, the results should be viewed in light of some possible limitations. First, the use of a prevalidated DMA instrument introduces uncertainty regarding the extent to which the construct of data maturity and its associated domains were accurately and comprehensively measured. Although the domains included are conceptually aligned [14,15], the appropriateness and sufficiency of individual items have not yet been examined. However, the concurrent use of consensus meetings alongside the administration of the DMA allowed for contextual enrichment, thereby strengthening the interpretation of the results [45]. On the other hand, the use of consensus scoring may have reduced the visibility of disagreement among stakeholders. While this approach supported the development of a shared assessment, minority viewpoints and differences in experience may not have been fully reflected in the final scores. Yet, given the organizational focus of the DMA and the interprofessional nature of the CoP, reaching consensus enabled stakeholders to collectively verify whether specific policies, processes, or data-related practices were actually present within the organization. Second, the relatively small sample size and stakeholders being part of the CoPs may have introduced selection bias. Stakeholders were likely more engaged with, knowledgeable about, or interested in data-informed care than other stakeholders within the participating LTC organizations. As a result, the findings should be interpreted as exploratory and may not be fully transferable to other LTC organizations or the wider Dutch LTC sector [46].

For future research, longitudinal studies are needed to examine how LTC organizations evolve in their data maturity over time and how this influences the introduction of data-informed care. As an initial step toward strengthening data maturity, organizations may benefit from demonstrating the value of routinely collected EHR data through small-scale, practice-based projects. Such initiatives can provide tangible insights into care processes and increase stakeholder engagement. For example, by demonstrating the added value of data insights derived from routinely collected data (eg, through dashboards or management information), organizations may increase awareness of the potential of data-informed care, strengthen stakeholder engagement, and foster organizational support for the broader implementation of data-informed care. Alternatively, organizations may choose to focus on strengthening data governance by developing clear governance structures, responsibilities, and roles for data management and use. Such initiatives may facilitate collaboration between care professionals, managers, and data and IT specialists, thereby bridging the gap between clinical practice and technical expertise. Moreover, future research should include larger, multisite studies with a broader range of organizations and stakeholders to capture a representative sample of LTC organizations to enhance the generalizability of findings. The authors are currently engaged in the ongoing validation of the DMA, in collaboration with the developers, with the aim of improving the DMA into a validated instrument to assess data maturity in health care organizations. Ideally, this will lead to the development of a gold standard that can be adopted and supported internationally.

### Conclusions

This exploratory mixed methods study provides insight into data maturity within 3 LTC organizations, suggesting that overall data maturity currently remains low across the participating organizations. Findings highlight not only low foundations in strategy, governance, and data quality but also reveal gaps in leadership and culture regarding data that constrain organizational learning and sustainable data use. Advancing toward data-informed care, in which quantitative data are combined with qualitative insights, including the experiences and knowledge of clients, their relatives, and care professionals, requires integrated strategies that go beyond strengthening technical infrastructures and cultural conditions, underscoring the importance of a shared vision and interprofessional collaboration among stakeholders, while considering LTC context-specific approaches.

## Abbreviations

AWO-L: Academische Werkplaats Ouderenzorg Limburg
BI: business intelligence
CoP: community of practice
DAMA-DMBOK: Data Management Body of Knowledge
DMA: data maturity assessment
EHR: electronic health record
FAIR: Findable, Accessible, Interoperable, and Reusable
FHML: Faculty of Health, Medicine and Life Sciences Research Ethics Committee
LTC: long-term care

## References

[1] Sanford AM, Orrell M, Tolson D (2015). An international definition for “nursing home”. J Am Med Dir Assoc.

[2] Cammer A, Morgan D, Stewart N (2014). The hidden complexity of long-term care: how context mediates knowledge translation and use of best practices. Gerontologist.

[3] Nolan M, Brown J, Davies S, Nolan J, Keady J (2006). The senses framework: improving care for older people through a relationship-centred approach. https://shura.shu.ac.uk/280/1/PDF_Senses_Framework_Report.pdf.

[4] Kruse CS, Goswamy R, Raval Y, Marawi S (2016). Challenges and opportunities of big data in health care: a systematic review. JMIR Med Inform.

[5] Fijten R, Wee L, Dekker A, Roumen C (2021). Data-driven shared decision-making: a paradigm shift. J Radiat Oncol Inform.

[6] Bouchmal S, Sion KY, Hamers JP, Aarts S (2025). Toward data-informed care in long-term care: qualitative analysis. JMIR Aging.

[7] Abdul-Azeez O, Ihechere AO, Idemudia C (2024). Enhancing business performance: the role of data-driven analytics in strategic decision-making. Int j manag entrep res.

[8] Mettler T, Vimarlund V (2009). Understanding business intelligence in the context of healthcare. Health Informatics J.

[9] Negash S, Gray P, Burstein F, Holsapple CW (2008). Handbook on Decision Support Systems 2: Variations.

[10] Evans JM, Grudniewicz A, Baker GR, Wodchis WP (2016). Organizational context and capabilities for integrating care: a framework for improvement. Int J Integr Care.

[11] Çömlek O (2025). The effects of organizational learning capacity on innovative performance of healthcare organizations. Hamidiye Med J.

[12] van Steenbergen M, Bos R, Brinkkemper S, van de Weerd I, Bekkers W, Winter R, Zhao JL, Aier S (2010). Global Perspectives on Design Science Research5th International Conference, DESRIST 2010, St Gallen, Switzerland, June 4-5, 2010 Proceedings.

[13] Lahrmann G, Marx F, Winter R, Zhao JL, Aier S (2010). Global Perspectives on Design Science Research5th International Conference, DESRIST 2010, St Gallen, Switzerland, June 4-5, 2010 Proceedings.

[14] Al-Sai ZA, Husin MH, Syed-Mohamad SM (2023). Big data maturity assessment models: a systematic literature review. Big Data Cogn Comput.

[15] van den Berg B, Lukkien D, de Charro F, Snellen M, Beelen A, Bouwens M (2024). Op welke manier kan datagedreven werken bijdragen aan meer doelmatigheid en kwaliteit in de langdurige zorg?. TSG-Tijdschr gezondheidswet.

[16] Proença D, Borbinha J, Méndez E, Crestani F, Ribeiro C, David G, Lopes JC (2018). Digital Libraries for Open Knowledge: 22nd International Conference on Theory and Practice of Digital Libraries, TPDL 2018, Porto, Portugal, September 10–13, 2018, Proceedings.

[17] Fetters MD, Curry LA, Creswell JW (2013). Achieving integration in mixed methods designs-principles and practices. Health Serv Res.

[18] Campbell S, Greenwood M, Prior S (2020). Purposive sampling: complex or simple? Research case examples. J Res Nurs.

[19] Lave J, Wenger E (1991). Situated Learning: Legitimate Peripheral Participation.

[20] Hydén LC, Bülow PH (2003). Who’s talking: drawing conclusions from focus groups—some methodological considerations. Int J Soc Res Methodol.

[21] Kitzinger J (1995). Qualitative research. Introducing focus groups. BMJ.

[22] Hacking C, Verbeek H, Hamers JPH, Aarts S (2023). The development of an automatic speech recognition model using interview data from long-term care for older adults. J Am Med Inform Assoc.

[23] Kuckartz U, Rädiker S (2019). Analyzing Qualitative Data with MAXQDA.

[24] Tesch R (2013). Qualitative Research: Analysis Types and Software.

[25] Xu W, Zammit K (2020). Applying thematic analysis to education: a hybrid approach to interpreting data in practitioner research. Int J Qual Methods.

[26] Khandkar SH (2009). Open coding. https://pages.cpsc.ucalgary.ca/~saul/wiki/uploads/CPSC681/open-coding.pdf?utm_source.

[27] Corbin JM, Strauss AL (2014). Basics of Qualitative Research: Techniques and Procedures for Developing Grounded Theory.

[28] Shenton AK (2004). Strategies for ensuring trustworthiness in qualitative research projects. Educ Inf.

[29] Wang Y, Kung L, Byrd TA (2018). Big data analytics: understanding its capabilities and potential benefits for healthcare organizations. Technol Forecast Soc Change.

[30] Valentino L, Hozak MA, Nelson JW, Felgen J, Hozak MA (2021). Using Predictive Analytics to Improve Healthcare Outcomes.

[31] Singer SJ, Benzer JK, Hamdan SU (2015). Improving health care quality and safety: the role of collective learning. J Healthc Leadersh.

[32] Cilliers F, Greyvenstein H (2012). The impact of silo mentality on team identity: an organisational case study. SA J Ind Psychol.

[33] Berndtsson M, Ericsson A, Svahn T (2020). Scaling up data‐driven pilot projects. AI Mag.

[34] Bernardo BMV, Mamede HS, Barroso JMP, dos Santos VMPD (2024). Data governance & quality management—innovation and breakthroughs across different fields. J Innov Knowl.

[35] Khatri V, Brown CV (2010). Designing data governance. Commun ACM.

[36] Data Management Association (2017). DAMA-DMBOK: Data Management Body of Knowledge.

[37] Chakravorty R (2020). Common challenges of data governance. J Secur Oper Custody.

[38] Greene SM, Reid RJ, Larson EB (2012). Implementing the learning health system: from concept to action. Ann Intern Med.

[39] Foley T, Vale L (2023). A framework for understanding, designing, developing and evaluating learning health systems. Learn Health Syst.

[40] Friedman CP, Lomotan EA, Richardson JE, Ridgeway JL (2024). Socio-technical infrastructure for a learning health system. Learn Health Syst.

[41] (2022). Towards an integrated health information system in the Netherlands. https://www.oecd.org/content/dam/oecd/en/publications/reports/2022/02/towards-an-integrated-health-information-system-in-the-netherlands_72e82a0a/a1568975-en.pdf.

[42] Hussein R, Scherdel L, Nicolet F, Martin-Sanchez F (2023). Towards the European Health Data Space (EHDS) ecosystem: a survey research on future health data scenarios. Int J Med Inform.

[43] Llena-Nozal A, Barszczewski J, Rauet-Tejeda J (2025). How do countries compare in their design of long‑term care provision? A typology of long-term care systems. https://www.oecd.org/content/dam/oecd/en/publications/reports/2025/06/how-do-countries-compare-in-their-design-of-long-term-care-provision_035a4e96/44f5453a-en.pdf.

[44] Österle A, Powell M, Agartan TI, Béland D (2024). Research Handbook on Health Care Policy.

[45] Thurmond VA (2001). The point of triangulation. J Nurs Scholarsh.

[46] Smith LH (2020). Selection mechanisms and their consequences: understanding and addressing selection bias. Curr Epidemiol Rep.

